# Isolated Superior Mesenteric Arterial Dissection: A Rare Case

**DOI:** 10.7759/cureus.84534

**Published:** 2025-05-21

**Authors:** Prabina Basnet, Sudeep Chapagain, Surendra Khanal, Prince Darko, Nimeshi Fernando

**Affiliations:** 1 Internal Medicine, Piedmont Athens Regional, Athens, USA

**Keywords:** abdominal pain, bp goal, ct abdomen, isolated spontaneous superior mesenteric artery dissection, life-threatening

## Abstract

Isolated spontaneous superior mesenteric artery dissection (ISMAD) is an extremely rare condition with a wide range of vague clinical presentations. Notably, ISMAD is a very uncommon cause of abdominal pain and can be fatal, resulting in life-threatening complications. Therefore, this condition should not be missed and appropriately managed. Here, we report the case of a 51-year-old male presenting with severe epigastric pain who was found to have superior mesenteric artery dissection on a CT scan of the abdomen. He was managed successfully with a conservative approach, which included bowel rest, beta-blockers, and anticoagulants.

## Introduction

Peripheral artery dissection, such as isolated spontaneous superior mesenteric artery dissection (ISMAD), is very rare. The increasing incidence of ISMAD is probably due to the increasing use and availability of imaging such as CT angiogram and MRI. Abdominal pain along with nausea and/or vomiting is a common presentation; however, it can be an incidental finding on imaging [1]. Presentation with chronic abdominal pain worsening after meals has also been reported. Rarely, it can present as life-threatening conditions such as acute bowel ischemia, gangrene, and aneurysmal rupture [1,2]. Although the majority of ISMAD cases are spontaneous, certain comorbidities, such as hypertension, atherosclerosis, and connective tissue diseases; certain vascular diseases, such as fibromuscular dysplasia; and medial degeneration are considered to be predisposing factors [3-5]. Prompt recognition and appropriate treatment lead to improved clinical outcomes [6]. Given the lack of evidence-based guidelines, this condition is managed similarly to the main artery dissection, which includes conservative treatment with bowel rest and anticoagulation and surgical treatment with endovascular stenting or open surgical repair [6,7].

## Case presentation

A 51-year-old male with a medical history of hypertension presented with severe epigastric pain for two hours. The pain was acute, deep, sharp, non-radiating, and progressing. He did not have nausea, vomiting, fever, or other gastrointestinal symptoms. On physical exam, blood pressure (BP) was 186/128 mmHg on the right arm with a normal heart rate. On abdominal examination, there was significant tenderness on deep palpation, with no rebound tenderness or guarding. Bowel sounds were normal, and no palpable mass was detected.

**Figure 1:**
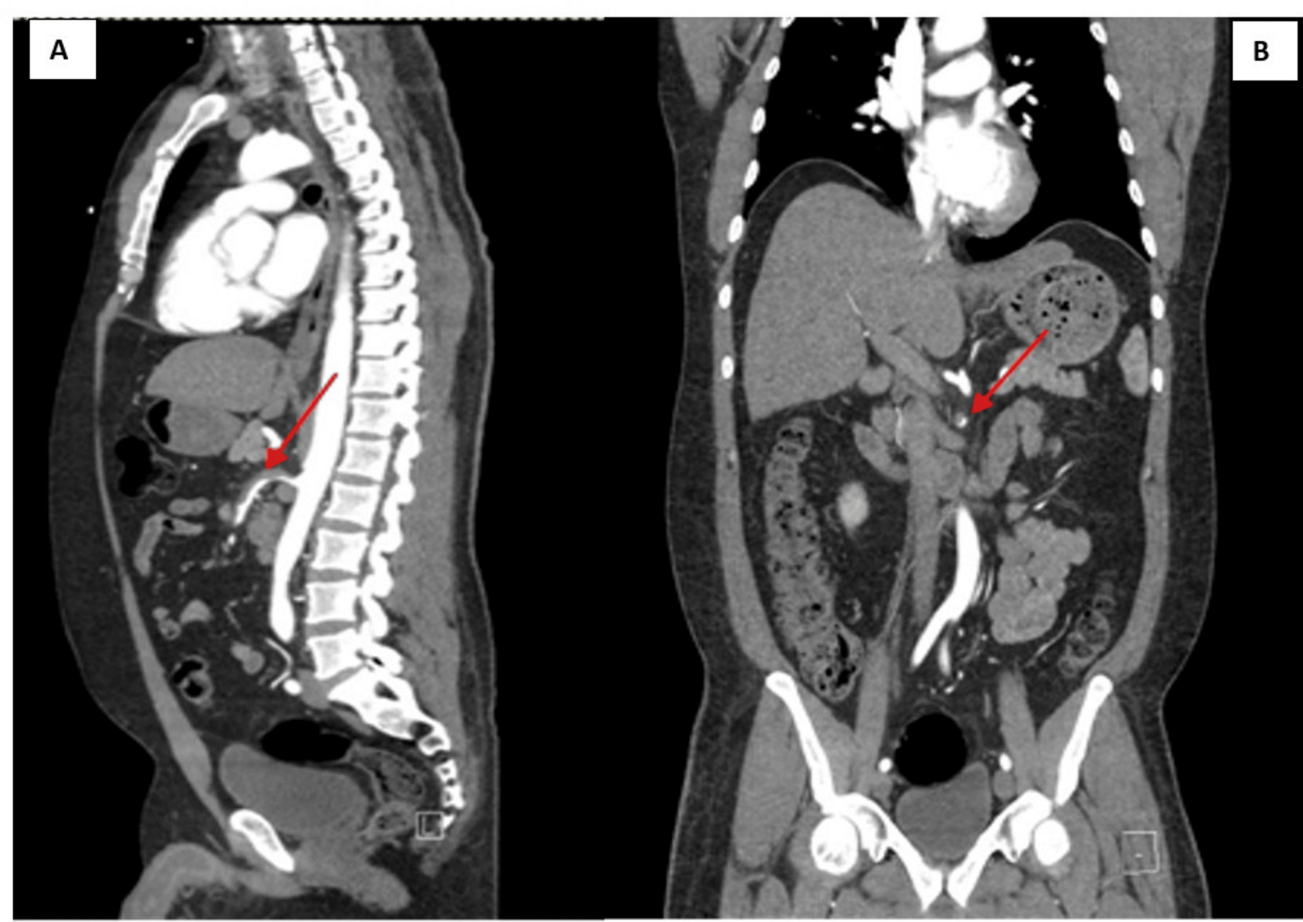
Sagittal (A) and coronal view (B) of CT angiogram of the abdomen and pelvis with superior mesenteric artery (SMA) dissection just after the aortic takeoff, demonstrating thrombus (red arrow) in the false lumen of the SMA dissection.

**Figure 2:**
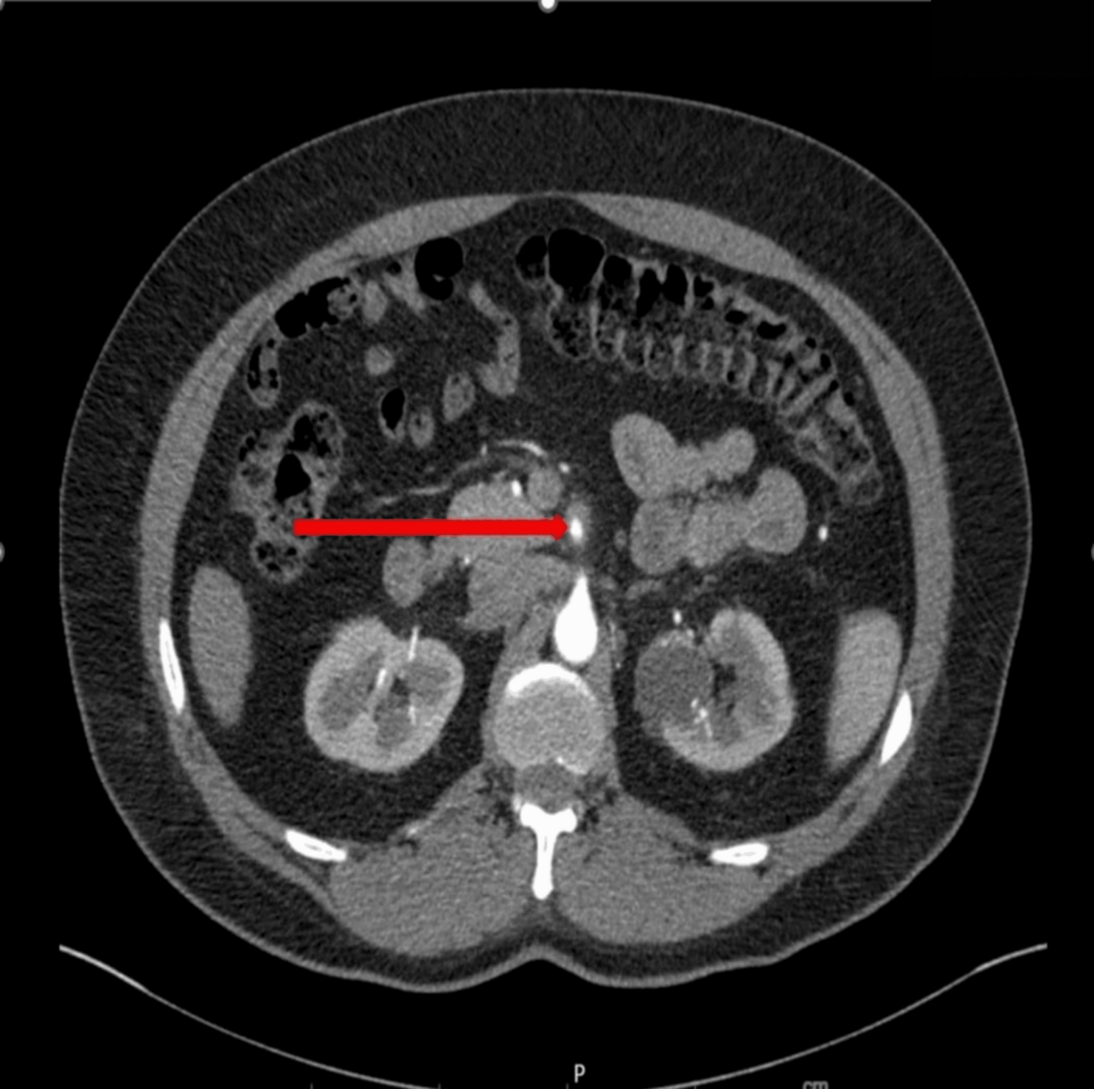
CT angiography of the abdomen and pelvis (axial section) showing superior mesenteric artery dissection with intramural hematoma.

The patient’s laboratory findings were unremarkable. As the patient presented with severe epigastric pain in the setting of uncontrolled hypertension, vascular causes, such as aortic dissection, were one of the differential diagnoses. Therefore, CT imaging was done. CT angiography of the abdomen and pelvis revealed a short-segment superior mesenteric artery (SMA) dissection without bowel ischemia (Figures 1, 2). Vascular surgery was also consulted to ensure expert evaluation and assess the need for surgical intervention. He was treated conservatively with intravenous (IV) esmolol drip, aspirin, statin, and IV heparin drip. Systolic BP goal of <120 mmHg and heart rate of <60 beats/minute was met with a titrating dose of esmolol drip. His epigastric pain gradually resolved with conservative measures. A repeat CT of the abdomen obtained after 48 hours showed no progression of the dissection. The patient was discharged on aspirin, apixaban, atorvastatin, carvedilol, and losartan with a plan to repeat imaging in six weeks.

## Discussion

Main artery dissections extending to the peripheral artery can occur frequently, such as aortic trunk dissection extending to the SMA. However, only as few as 3,000 cases of ISMAD have been reported worldwide [8]. The low incidence might be due to its rarity, vague presentation, and limited availability of imaging. The recent increase in the detection of ISMAD is probably due to the increasing use of advanced imaging modalities [4,8-10].

ISMAD can have a wide range of varying clinical presentations from asymptomatic incidental findings in the imaging to abdominal pain to life-threatening emergencies such as acute bowel ischemia and gangrene or arterial rupture [1,3]. Therefore, this rare condition should not be missed. The most common symptoms are abdominal pain and distension, back pain, nausea, vomiting, and per rectum bleeding [10]. Although the majority of ISAMD cases are spontaneous, certain risk factors, such as hypertension, atherosclerosis, vasculitis, and connective tissue diseases, are considered to be predisposing factors [3]. Other conditions, such as previous abdominal surgery, diabetes, or trauma, have been reported to be associated with SMA dissection [3]. Our patient presented only with an acute onset of severe abdominal pain and had a history of uncontrolled hypertension, non-adherent to medication. His BP was also elevated at presentation, which could have contributed to the problem. The pathophysiology is thought to be similar to the other arterial dissections, i.e., blood seeps within the medial and adventitial layers and propagates along the way [1,3]. Contrast-enhanced CT scan and MRI are preferred imaging modalities as they help classify the dissection and look into other abdominal organs to rule out other differential diagnoses. MRI can be used in patients with contrast allergy. CT angiography is the gold standard for the diagnosis as it better demonstrates thrombosis, double lumen (the most classical sign), and dissecting flap [1,2,8,10].

There are limited evidence-based guidelines for its management [1,11]. The options include conservative treatment and a revascularization approach guided by the hemodynamic stability and signs of complications on imaging. The two revascularization techniques are endovascular and open surgical repair [1,12]. ISMAD is managed similarly to aortic dissection, as there is no standard guideline. Conservative therapy includes antithrombotic therapy, pain management, BP control, and anticoagulant therapy. The main aim of antithrombotic therapy is to prevent vessel thrombosis and reduce the likelihood of progression. Immediate revascularization is indicated in hemodynamically unstable patients with signs and symptoms of ischemia and refractory pain. Radiological evidence of progression or worsening dissection, such as formation of thrombus, narrowing, or saccular aneurysm formation, is also an indication of immediate revascularization [13]. Repeat imaging is obtained in 48 hours to monitor for the progression of the dissection as well as complications. If found to be stable, patients are transitioned to oral anticoagulants with close follow-up with vascular surgery and interval imaging for monitoring. Complete SMA remodeling is common in patients managed conservatively. For patients managed conservatively, clinical follow-up is recommended in the first month and then every year until complete mesenteric remodeling has occurred or for a maximum of two years if no changes are noted [7]. In our patient, follow-up was planned at six weeks with vascular surgery.

## Conclusions

ISMAD is a rare, potentially life-threatening vascular condition. The outcome is mostly favorable with conservative treatment, which includes anticoagulation and BP control. Rarely, surgical intervention is required. Our case demonstrates a patient presenting with severe epigastric pain who was found to have ISMAD on the CT of the abdomen. Despite being considered a rare condition, we expect that ISMAD will be detected more frequently with the increasing use of advanced imaging modalities. Although it may present with vague symptoms, physicians should consider ISMAD as one of the differentials for abdominal pain. Our patient’s condition was managed conservatively, resulting in the resolution of the symptoms. There was no further progression while monitoring with repeat imaging as well. Therefore, early recognition of symptoms is important for timely diagnosis and appropriate management to ensure a favorable outcome.
